# Supplementary material for: Quantifying gender bias towards politicians in cross-lingual language models
Source: PLoS One. 2023 Nov 28;18(11):e0277640. doi: 10.1371/journal.pone.0277640 (PMC10684026; doi:10.1371/journal.pone.0277640)
Supplement: S1 Text — (PDF) [file pone.0277640.s001.pdf]

S1 Text. Politician gender.

In Tab 1, we list genders classified as non-binary gender. We present detailed counts on all gender categories for each of the analyzed languages in Tab 2.

Non-binary gender
genderfluid
genderqueer
non-binary
third gender
transfeminine
transgender female
transgender male

Table 1. List of genders grouped together as non-binary.

	Languages						
Gender	Arabic	Chinese	English	French	Hindi	Russian	Spanish
male	206.526	207.713	206.493	233.598	206.778	208.982	226.492
female	44.960	45.681	44.701	53.435	44.956	45.275	50.886
unknown	2.268	8.341	2.291	2.330	2.282	2.274	2.462
transgender female	55	55	52	55	55	55	55
transgender male	4	4	4	4	4	4	4
non-binary	4	4	4	4	4	4	4
cisgender female	2	2	2	2	2	2	2
genderfluid	1	1	1	1	1	1	1
genderqueer	1	1	0	1	1	1	1
female organism	1	1	1	1	1	1	1
male organism	1	1	1	1	1	1	1
third gender	1	1	1	1	1	1	1
transfeminine	1	1	1	1	1	1	1

Table 2. Counts of politicians grouped by gender based on Wikidata information. Numbers across languages differ due to politician data not being available in all languages.
