# Supplementary material for: Quantifying gender bias towards politicians in cross-lingual language models
Source: PLoS One. 2023 Nov 28;18(11):e0277640. doi: 10.1371/journal.pone.0277640 (PMC10684026; doi:10.1371/journal.pone.0277640)
Supplement: S2 Text — (PDF) [file pone.0277640.s002.pdf]

S2 Text. Supersenses.

We list the word senses as defined for adjectives in [1] and for verbs in [2].

Supersense	Example Words
Behavior	bossy, deceitful, talkative, tame, organized, adept, popular
Body	alive, athletic, muscular, ill, deaf, hungry, female
Feeling	angry, embarrassed, willing, pleasant, cheerful
Mind	clever, inventive, silly, educated, conscious
Misc.	important, chaotic, affiliated, equal, similar, vague
Motion	gliding, flowing, immobile
Perception	purple, shiny, taut, glittering, smellier, salty, noisy
Quantity	billionth, enough, inexpensive, profitable
Social	affluent, upscale, military, devout, Asian, arctic, rural
Spatial	compact, gigantic, circular, hollow, adjacent, far
Substance	creamy, frozen, dense, moist, ripe, closed, metallic, dry
Temporal	old, continual, delayed, annual, junior, adult, rapid
Weather	rainy, balmy, foggy, hazy, humid

Table 1. List of supersenses for adjectives as defined in [1].

Supersense	Example Words
Body	blink, blush, injure
Change	augment, complicate, disappear, mature
Cognition	analyze, know, memorize, omit
Communication	alert, cite, forbid, propose
Competition	conquer, enlist, overcome, protect
Consumption	dine, eat, want, starve
Contact	carve, fasten, grasp, launch
Creation	decorate, invent, motivate
Emotion	annoy, despise, frighten, mourn
Motion	arrive, intersect, lunge, negotiate,
Perception	behold, creak, detect, monitor
Possession	accord, locate, own, pretend
Social	dare, mary, obey, preside, tolerate
Stative	contain, occupy, lurk, underlie
Weather	blaze, glare, plague, spark

Table 2. List of supersenses for verbs as defined in [2].

References

1. Tsvetkov Y, Schneider N, Hovy D, Bhatia A, Faruqui M, Dyer C. Augmenting English Adjective Senses with Supersenses. In Proceedings of the Ninth International Conference on Language Resources and Evaluation. Reykjavik, Iceland: European Language Resources Association. 2014;4359–4365. Available

from:
http://www.lrec-conf.org/proceedings/lrec2014/pdf/1096_Paper.pdf.

2. Miller GA, Leacock C, Tengi R, Bunker RT. A Semantic Concordance. In Human Language Technology: Proceedings of a Workshop Held at Plainsboro, New Jersey, March 21-24, 1993. 1993. Available from:
https://www.aclweb.org/anthology/H93-1061.
