# Supplementary material for: Quantifying gender bias towards politicians in cross-lingual language models
Source: PLoS One. 2023 Nov 28;18(11):e0277640. doi: 10.1371/journal.pone.0277640 (PMC10684026; doi:10.1371/journal.pone.0277640)
Supplement: S3 Text — (PDF) [file pone.0277640.s003.pdf]

S3 Text. Additional Experiments.

We conducted three additional experiments in which we analyzed gender bias towards 1) politicians whose country of origin (*i.e.*, their nationality) uses the respective language as an official language, 2) the most popular politicians for each language, and 3) politicians born before and after Baby Boom (1946) to control for temporal changes.

Native language analysis In the following, we analyzed words generated based on a smaller subset of politicians. In particular, for each language, we examined terms associated with politicians whose country of origin (*i.e.*, their nationality) uses the respective language as an official language. To this end, we queried Wikidata for the relevant nationality data and relied on Wikipedia for the list of countries using the analyzed languages as official languages.

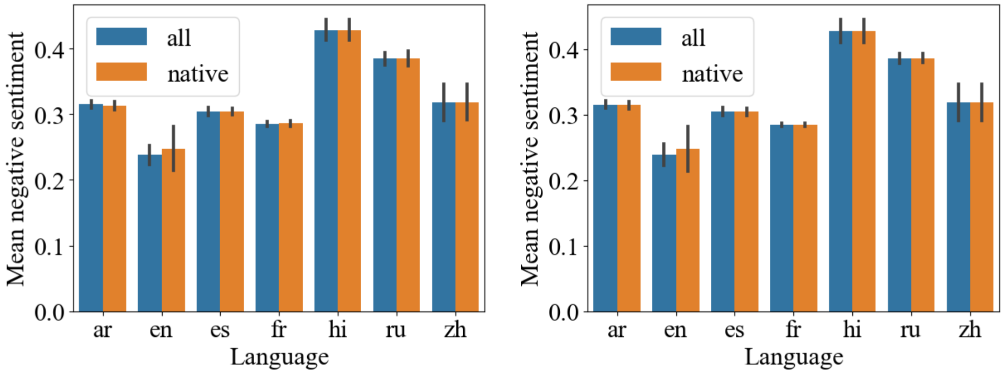

Fig 1. Mean negative sentiment of words associated with politicians whose country of origin uses the language as an official language for each language and male (left) and female (right) politicians for each language averaged over analyzed language models.

We assumed that in this restricted setup language models were more often exposed to the particular politician names and thus have encoded a certain level of bias towards these entities. In general, we did not find differences in negative sentiment (see Fig 1) towards politicians native to the language. Only for English, we observed a tendency to higher negativity towards native politicians. However, this might be owed to the fact that politicians from the Anglosphere are more well-known than their colleagues from countries outside of the English-speaking world.

Popularity analysis Prior work posits that a pre-trained model might learn to associate negativity with an NE if a name is often mentioned in negative linguistic contexts [1]. This might be the case, especially for the most popular politicians in our dataset. Therefore, in order to control for the effect of popularity, we separately investigated words associated with the most well-known politicians. We used the number of times a politician was mentioned on Wikipedia in all articles as a proxy for popularity. For each language, we selected 10k most famous politicians and compared generated words on these subsets to the results obtained on the whole dataset.

As presented in Fig 2, we did not find differences in negative sentiment towards the most famous politicians. We hypothesize that this is due to the fact that most of the data multilingual language models were pre-trained on comes from Wikipedia, a data

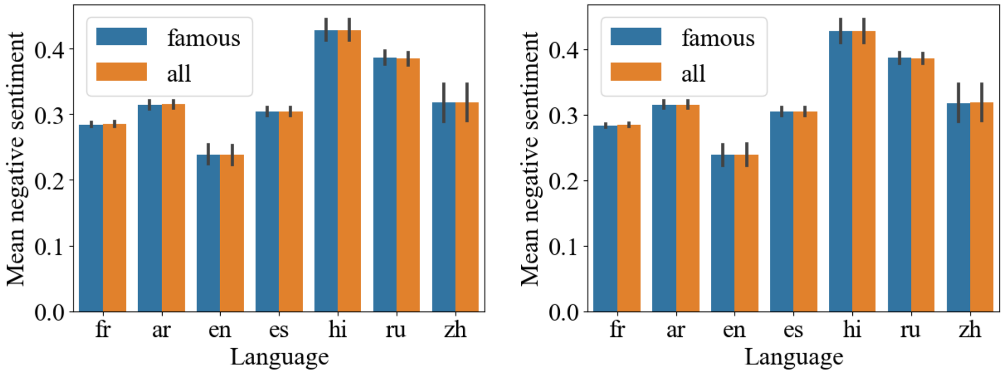

Fig 2. Mean negative sentiment of words associated with the most popular 10k politicians for each language and male (left) and female (right) politicians for each language averaged over analyzed language models.

source where informative language is used. We conjecture that differences in the mean negative sentiment are due to differences across languages, and to a smaller extent depend on the model architecture.

Temporal analysis In the final set of experiments, we analyzed differences in associations language models make for politicians dependent on their birth year. (We queried only names of politicians born from the 20th century onward. We assumed this restriction decreases temporal influences with respect to politicians' descriptions.) To test this hypothesis, we analyzed words associated with politicians born roughly in the first vs. in the second half of the 20th century. To this end, we queried the date of birth for each politician included in our dataset, and compared words generated for politicians born before and after the 1st of January 1946. We decided to use 1946 as a cutoff since it marks the first year after World War II and starts a period of the Western world's history popularly called the mid-20th century Baby Boom which is considered the most impactful generation shift in history [2].

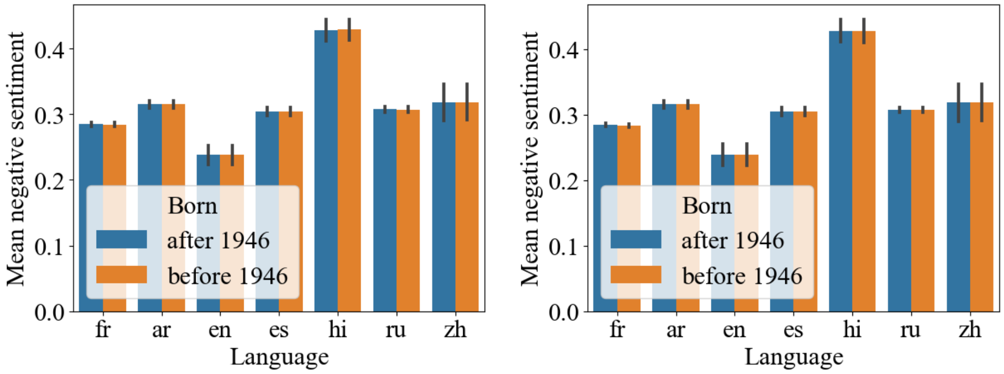

Fig 3. Mean negative sentiment of words associated with politicians born before and after 1946 for male (left) and female (right) politicians for each language averaged over analyzed language models.

We tested whether the sentiment towards politicians differs when we analyze separately politicians born before and after 1946 in Fig 3. Again, we did not see any differences across languages. This finding confirms our hypothesis that filtering out politicians born from the 20th century onward decreases temporal effects.

References

1. Prabhakaran V, Hutchinson B, Mitchell M. Perturbation Sensitivity Analysis to Detect Unintended Model Biases. In Proceedings of the 2019 Conference on Empirical Methods in Natural Language Processing and the 9th International Joint Conference on Natural Language Processing. Hong Kong, China: Association for Computational Linguistics. 2019;5740–5745. Available from: `https://www.aclweb.org/anthology/D19-1578`.

2. Van Bavel J, Reher DS. The Baby Boom and Its Causes: What We Know and What We Need to Know. Population and Development Review. 2013:39:257–288. `https://doi.org/10.1111/j.1728-4457.2013.00591.x`.
