# Supplementary material for: Quantifying gender bias towards politicians in cross-lingual language models
Source: PLoS One. 2023 Nov 28;18(11):e0277640. doi: 10.1371/journal.pone.0277640 (PMC10684026; doi:10.1371/journal.pone.0277640)
Supplement: S1 Table — (PDF) [file pone.0277640.s004.pdf]

S1 Table. Word Orderings.

We list the word orderings used for the analyzed languages in Tab 1.

Language	Order of Subject, Object and Verb	Order of Adjective and Noun
Arabic	VSO	Noun Adj
Chinese	SVO	Adj Noun
English	SVO	Adj Noun
French	SVO	Noun Adj
Hindi	SOV	Adj Noun
Russian	SVO	Adj Noun
Spanish	SVO	Noun Adj

Table 1. List of word orderings we follow during the language generation process based on the World Atlas of Language Structures [1, 2].

References

1. Dryer MS. Order of Adjective and Noun. In Dryer MS, Haspelmath M, editors. The World Atlas of Language Structures Online. Leipzig: Max Planck Institute for Evolutionary Anthropology. 2013. Available from: `https://wals.info/chapter/87`.

2. Dryer MS. Order of Subject, Object, and Verb. In Dryer MS, Haspelmath M, editors. The World Atlas of Language Structures Online. Leipzig: Max Planck Institute for Evolutionary Anthropology. 2013. Available from: `https://wals.info/chapter/81`.
