# Supplementary material for: Quantifying gender bias towards politicians in cross-lingual language models
Source: PLoS One. 2023 Nov 28;18(11):e0277640. doi: 10.1371/journal.pone.0277640 (PMC10684026; doi:10.1371/journal.pone.0277640)
Supplement: S2 Table — (PDF) [file pone.0277640.s005.pdf]

## S2 Table. Sentiment Analysis Evaluation.

In Tab 1, we present an evaluation of the sentiment lexica in a text classification task on a selected dataset for each of the languages. We use the resulting lexica to automatically label instances with their sentiment, based on the average sentiment of words in each sentence. The sentiment lexicon approach achieves comparable performance to a supervised model for most of the analyzed languages.

| Language | Dataset | Number of texts | Self-supervised SentiVAE-based | Supervised Model |
|---|---|---|---|---|
| Arabic | Elnagar et al. [1] | 93 700 | 82.7 ($F1$) | 81.6 ($F1$) |
| Chinese | Zhang and Chen [2] | 30 000 | 78.2 ($F1$) | 87.12 ($F1$) |
| French | Blard [3] | 200 000 | 72.8 ($F1$) | 97.36 ($F1$) |
| Hindi | Kunchukuttan et al. [4] | 4 705 | 63.5 (*Acc.*) | 75.71 (*Acc.*) |
| Russian | Shalkarbayuli et al. [5] | 8 263 | 67.0 ($F1$) | 70.00 ($F1$) |
| Spanish | Díaz-Galian et al. [6] | 1 474 | 54.8 ($F1$) | 50.7 ($F1$) |

**Table 1.** Classification performance on the respective test sets for a self-supervised approach using SentiVAE sentiment lexica vs. the best-reported result in the paper presenting the respective dataset for each language. Performance metric given in the brackets.

## References


1. Elnagar A, Khalifa YS, Einea A. Hotel Arabic-Reviews Dataset Construction for Sentiment Analysis Applications. Shaalan K, Hassanien AE, Tolba F. Cham: Springer International Publishing. 2018. Available from: https://doi.org/10.1007/978-3-319-67056-0_3.

2. Zhang L, Chen C. Sentiment Classification with Convolutional Neural Networks: An Experimental Study on a Large-Scale Chinese Conversation Corpus. 12th International Conference on Computational Intelligence and Security. 2016;165–169. Available from: https://ieeexplore.ieee.org/stamp/stamp.jsp?tp=&arnumber=7820437.

3. Blard T. French sentiment analysis with BERT; 2020. Available from: https://github.com/TheophileBlard/french-sentiment-analysis-with-bert.

4. Kunchukuttan A, Kakwani D, Golla S, C GN, Bhattacharyya A, Khapra MM, Kumar P. AI4Bharat-IndicNLP Corpus: Monolingual Corpora and Word Embeddings for Indic Languages. arXiv preprint arXiv:200500085. 2005. Available from: https://arxiv.org/abs/2005.00085.

5. Shalkarbayuli A, Kairbekov A, Amangeldi Y. Comparison of traditional machine learning methods and Google services in identifying tonality on Russian texts. Journal of Physics: Conference Series. 2018;1117:012002. Available from: https://iopscience.iop.org/article/10.1088/1742-6596/1117/1/012002.

6. Díaz-Galiano MC, Vega M, Casasola E, Chiruzzo L, Cumbreras MÁG, Martínez-Cámara E, Moctezuma D, Montejo-Ráez A, Sobrevilla Cabezudo MA, Tellez E, Graff M, Miranda S. Overview of TASS 2019: One More Further for the Global Spanish Sentiment Analysis Corpus. In Proceedings of the Iberian

Languages Evaluation Forum. 2019;550–560. Available from: http://ceur-ws.org/Vol-2421/TASS_overview.pdf.
