# Supplementary material for: Quantifying gender bias towards politicians in cross-lingual language models
Source: PLoS One. 2023 Nov 28;18(11):e0277640. doi: 10.1371/journal.pone.0277640 (PMC10684026; doi:10.1371/journal.pone.0277640)
Supplement: S3 Table — (PDF) [file pone.0277640.s006.pdf]

S3 Table. Generated Words.

We present detailed counts of generated adjectives in Tab 1.

Sentiment	m-bert-cased		m-bert-uncased		xlm-base		xlm-large		xlm-r-base		xlm-r-large	
	male	*fem*	*male*	*fem*	*male*	*fem*	*male*	*fem*	*male*	*fem*	*male*	*fem*
Arabic	199	199	169	169	694	680	287	286	386	370	311	283
Chinese	53	53	53	53	319	317	149	149	404	363	407	388
English	714	628	801	615	941	773	642	574	369	284	368	324
French	356	329	249	222	666	594	255	252	272	250	301	281
Hindi	85	85	30	30	183	183	130	130	343	314	345	307
Russian	206	206	171	171	479	466	147	147	265	247	279	255
Spanish	485	484	432	426	1031	1026	403	403	481	470	481	469

Table 1. Unique counts of lemmatized adjectives generated by each language model for each language grouped by gender. In the language generation process, we retrieve the top 100 adjectives with the highest probability for all languages but English where we select the top 20 adjectives.
