# Supplementary material for: Quantifying gender bias towards politicians in cross-lingual language models
Source: PLoS One. 2023 Nov 28;18(11):e0277640. doi: 10.1371/journal.pone.0277640 (PMC10684026; doi:10.1371/journal.pone.0277640)
Supplement: S4 Table — (PDF) [file pone.0277640.s007.pdf]

S4 Table. Cluster Analysis.

We present the results of the cluster analysis for Russian in Tab 1.

Cluster	Example words
1	first, summer, official, most
2	small, big, main, best, leading
3	another, international, average, young
4	lower, short, west, northern, oriental
5	white, old, pretty, green, gold

Table 1. Results of the cluster analysis for Russian (translated into English) for words generated with XLM-base in association with male politicians. We list five words from every cluster.
