# Supplementary material for: Quantifying gender bias towards politicians in cross-lingual language models
Source: PLoS One. 2023 Nov 28;18(11):e0277640. doi: 10.1371/journal.pone.0277640 (PMC10684026; doi:10.1371/journal.pone.0277640)
Supplement: Attachment — Submitted filename: PloS One - Reviews.pdf [file pone.0277640.s008.pdf]

Dear PLOS ONE Academic Editor,

We would like to start by thanking the reviewers for their time and consideration in evaluating our work, and we are writing to ask you to examine a revised version of our manuscript. As a summary, our paper explores a simple method to probe pre-trained language models for gender bias, which we use to effect a multi-lingual study of gender bias towards politicians. We construct a dataset of 250k politicians from most countries in the world and quantify adjective and verb usage around those politicians' names as a function of their gender. We conduct our study in 7 languages across 6 different language modeling architectures.

In the revised version of the paper, we incorporate the reviewers' feedback and add additional experiments, where we conduct a more in-depth analysis of gender bias towards politicians in multilingual pre-trained language models. We extend our experiments with three additional experimental setups: 1) We perform an analysis on a subset of the most popular politicians for each of the analyzed languages; 2) We conduct an analysis on a subset of names where for each language we examine words generated for politicians whose country of origin (i.e., their nationality) uses the respective language as an official language; 3) We split our dataset and analyze words generated for politicians born before and after Baby Boom (1946). We believe that these experiments enable analysis in a more controllable setup. However, we did not find any significant patterns. We include these results in the Appendix, while being open to moving them upon acceptance to the main body of the paper if the reviewers consider it beneficial for the paper.

Following feedback from reviewers, we have restructured the main body of the paper to increase its readability. We have expanded upon our definition of gender bias and type of bias analyzed. We have clarified our data generation process and have included an overview graph. Further, we have extended justification of our methodological choices in the experimental setup. We believe that these changes have increased the clarity of the paper.

Additionally, we have now made our dataset and code available in order to increase transparency and reproducibility of the paper and the conducted analysis. Since our approach can be extended to other types of entities, biases, and other languages, we hope that the presented methodology will initiate further studies of societal biases in large pre-trained language models. We include the list of politician names, and our code as an attachment to our submission.

Finally, we clarified in the attached cover letter that the funders had no role in study design, data collection and analysis, decision to publish, or preparation of the manuscript.

We thank the reviewers for the time and energy put into our submission. Below, we respond to the specific questions the reviews have raised inline and we mark our comments with a distinguished font and a different color -- blue.

PONE-D-22-03986
Quantifying Gender Bias Towards Politicians in Cross-Lingual Language Models
PLOS ONE

Dear Dr. Stanczak,

Thank you for submitting your manuscript to PLOS ONE. After careful consideration, we feel that it has merit but does not fully meet PLOS ONE's publication criteria as it currently stands. Therefore, we invite you to submit a revised version of the manuscript that addresses the points raised during the review process.

Please submit your revised manuscript by Jun 05 2022 11:59PM. If you will need more time than this to complete your revisions, please reply to this message or contact the journal office at plosone@plos.org. When you're ready to submit your revision, log on to https://www.editorialmanager.com/pone/ and select the 'Submissions Needing Revision' folder to locate your manuscript file.

Please include the following items when submitting your revised manuscript:

- A rebuttal letter that responds to each point raised by the academic editor and reviewer(s). You should upload this letter as a separate file labeled 'Response to Reviewers'.
- A marked-up copy of your manuscript that highlights changes made to the original version. You should upload this as a separate file labeled 'Revised Manuscript with Track Changes'.
- An unmarked version of your revised paper without tracked changes. You should upload this as a separate file labeled 'Manuscript'.

If you would like to make changes to your financial disclosure, please include your updated statement in your cover letter. Guidelines for resubmitting your figure files are available below the reviewer comments at the end of this letter.

If applicable, we recommend that you deposit your laboratory protocols in protocols.io to enhance the reproducibility of your results. Protocols.io assigns your protocol its own identifier (DOI) so that it can be cited independently in the future. For instructions see: https://journals.plos.org/plosone/s/submission-guidelines#loc-laboratory-protocols. Additionally, PLOS ONE offers an option for publishing peer-reviewed Lab Protocol articles, which describe protocols hosted on protocols.io. Read more information on sharing protocols at https://plos.org/protocols?utm_medium=editorial-email&utm_source=authorletters&utm_campaign=protocols.

We look forward to receiving your revised manuscript.

Kind regards,

Yang Xu, PhD

Academic Editor

PLOS ONE

Journal Requirements:

When submitting your revision, we need you to address these additional requirements.

1. Please ensure that your manuscript meets PLOS ONE's style requirements, including those for file naming. The PLOS ONE style templates can be found at

https://journals.plos.org/plosone/s/file?id=wjVg/PLOSOne_formatting_sample_main_body.pdf and

https://journals.plos.org/plosone/s/file?id=ba62/PLOSOne_formatting_sample_title_authors_affiliations.pdf

2. Thank you for stating the following financial disclosure:

“This work is partly funded by Independent Research Fund Denmark under grant

agreement number 9130-00092B.”

Please state what role the funders took in the study. If the funders had no role, please state: "The funders had no role in study design, data collection and analysis, decision to publish, or preparation of the manuscript."

If this statement is not correct you must amend it as needed.

Please include this amended Role of Funder statement in your cover letter; we will change the online submission form on your behalf.

3. Thank you for stating the following in the Acknowledgments Section of your manuscript:

“This work is mostly funded by Independent Research Fund Denmark under grant agreement number 9130-00092B.”

We note that you have provided additional information within the Acknowledgements Section that is not currently declared in your Funding Statement. Please note that funding information should not appear in the Acknowledgments section or other areas of your manuscript. We will only publish funding information present in the Funding Statement section of the online submission form.

Please remove any funding-related text from the manuscript and let us know how you would like to update your Funding Statement. Currently, your Funding Statement reads as follows:

“This work is partly funded by Independent Research Fund Denmark under grant

agreement number 9130-00092B.”

Please include your amended statements within your cover letter; we will change the online submission form on your behalf.

4. In your Data Availability statement, you have not specified where the minimal data set underlying the results described in your manuscript can be found. PLOS defines a study's minimal data set as the underlying data used to reach the conclusions drawn in the manuscript and any additional data required to replicate the reported study findings in their entirety. All PLOS journals require that the minimal data set be made fully available. For more information about our data policy, please see http://journals.plos.org/plosone/s/data-availability.

Upon re-submitting your revised manuscript, please upload your study’s minimal underlying data set as either Supporting Information files or to a stable, public repository and include the relevant URLs, DOIs, or accession numbers within your revised cover letter. For a list of acceptable repositories,

please see http://journals.plos.org/plosone/s/data-availability#loc-recommended-repositories. Any potentially identifying patient information must be fully anonymized.

Important: If there are ethical or legal restrictions to sharing your data publicly, please explain these restrictions in detail. Please see our guidelines for more information on what we consider unacceptable restrictions to publicly sharing data: http://journals.plos.org/plosone/s/data-availability#loc-unacceptable-data-access-restrictions. Note that it is not acceptable for the authors to be the sole named individuals responsible for ensuring data access.

We will update your Data Availability statement to reflect the information you provide in your cover letter.

5. Please include captions for your Supporting Information files at the end of your manuscript, and update any in-text citations to match accordingly. Please see our Supporting Information guidelines for more information: http://journals.plos.org/plosone/s/supporting-information.

Additional Editor Comments:

This paper provides a computational analysis of gender bias toward politicians in language models. Three expert reviewers have offered detailed comments on the manuscript. Although overall the reviewers agreed that the authors have made an attempt at a valuable problem, they pointed out many issues in the current version (most prominently, the lack of thoroughness in the exposition, and the extension and deepening of analyses) and provided extensive feedback on how to improve the manuscript in a major revision. The manuscript as it stands is not acceptable for publication at PLOS, and I encourage the authors to address the reviewer comments carefully in the revision.

R1 raises an issue about the input sources of the language models and controlling for the input. R1 also suggests a controlled analysis based on temporal considerations and raises a question about the discrepancy between English and other languages in the top adjectives generated.

To control for the generated words we restrict the Parts-of-Speech that are allowed and consider the word orderings that the analyzed languages follow. Moreover, we select solely top k words with the highest probability assigned by the models. Additionally, we have sampled generated words for manual evaluation. We already restricted our dataset to politicians born in XX century to decrease temporal influences. In an additional experiment, we have tested if there is a shift in sentiment toward politicians. We agree that there is a discrepancy between English and other languages. This, however, is due to the quality of the multilingual language models, while in this paper we focus on analyzing bias.

R2 raises a concern about the choice and relevance of politician names and suggests separate analyses to be performed on subsets of the names, tailored for individual languages. R2 and R1 indicate on the reviewer feedback form that the data underlying the findings have not been made fully available, which is contradictory to the authors' indication on the submission form — if this is a misunderstanding, please indicate clearly where the data can be accessed in the manuscript (and in the forthcoming response letter). It is also advised that the authors provide the link to the code repository that replicates all the analyses reported in the manuscript to be revised.

We include the list of politician names and our code as an attachment to our submission. Lists of generated words require a lot of storage space, therefore we do not include them for

now in our submission. However, we are open to uploading the data upon submission. We include a sample of our results in the current submission.

R3 suggests that the writing needs be substantially improved in terms of its structure, justification for the methodological choices (see R2's concurrent comment point #3., and R1's final comment), and correction for an overstatement about novel methodologies. R3 also points out correctly that the results presented seem surface explorations, and more in-depth analyses should be considered to address this issue in the cross-linguistic setting which include cross-language variability, reliability of methodology across languages and politicians. R2 concurs with this comment suggesting that the paper presented similar methodologies to an existing publication on gender word associates, but more analyses should be developed across languages, as well as those that provide better interpretation of the results and some correlational analyses on term frequencies.

We have restructured the paper and added more motivations for our methodological choices. Further, we have added additional experiments following the reviewers feedback. We believe that these changes have increased the quality of the paper.

[Note: HTML markup is below. Please do not edit.]

Reviewers' comments:

Reviewer's Responses to Questions

Comments to the Author

1. Is the manuscript technically sound, and do the data support the conclusions?

The manuscript must describe a technically sound piece of scientific research with data that supports the conclusions. Experiments must have been conducted rigorously, with appropriate controls, replication, and sample sizes. The conclusions must be drawn appropriately based on the data presented.

Reviewer #1: Yes

Reviewer #2: Yes

Reviewer #3: Partly

---

2. Has the statistical analysis been performed appropriately and rigorously?

Reviewer #1: Yes

Reviewer #2: Yes

Reviewer #3: Yes

---

3. Have the authors made all data underlying the findings in their manuscript fully available?

The PLOS Data policy requires authors to make all data underlying the findings described in their manuscript fully available without restriction, with rare exception (please refer to the Data Availability Statement in the manuscript PDF file). The data should be provided as part of the manuscript or its supporting information, or deposited to a public repository. For example, in addition to summary statistics, the data points behind means, medians and variance measures should be available. If there are restrictions on publicly sharing data—e.g. participant privacy or use of data from a third party—those must be specified.

Reviewer #1: No

Reviewer #2: No

Reviewer #3: Yes

---

4. Is the manuscript presented in an intelligible fashion and written in standard English?

PLOS ONE does not copyedit accepted manuscripts, so the language in submitted articles must be clear, correct, and unambiguous. Any typographical or grammatical errors should be corrected at revision, so please note any specific errors here.

Reviewer #1: Yes

Reviewer #2: Yes

Reviewer #3: No

---

5. Review Comments to the Author

Please use the space provided to explain your answers to the questions above. You may also include additional comments for the author, including concerns about dual publication, research ethics, or publication ethics. (Please upload your review as an attachment if it exceeds 20,000 characters)

Reviewer #1: The submitted work investigates the inherent gender biases learned by large contextualized neural language models (LMs) by probing sentiment biases of words that LMs associate with both male and female politicians. Distinct from previous work that attempts to quantify gender biases in language models, the authors consider a multi-lingual setting where both monolingual LMs and cross-lingual LMs are analyzed. Combined with previous methods in quantifying gender bias (PMI and latent variable model from Hoyle et al. (2019)), the authors propose a novel experimental setup that considers lexical sentiment scores of words that are predicted by the LMs to directly supersede or following the named entity of interest. Under the proposed experimental setup, gender biases in LMs are quantified by categorizing the list of words with the largest deviation w.r.t. gender, as well as statistical tests w.r.t. hyperparameters such as model size, model architecture, and language. The proposed methodology and experiment setup has the potential to be extended to analyze gender biases toward other types of named entities.

The authors promise to make the data fully available but I was not able to find the data in the materials submitted.

We include the list of politician names and our code as an attachment to our submission. Lists of generated words require a lot of storage space, therefore we do not include them for now in our submission. However, we are open to uploading the data upon submission. We include a sample of our results in the current submission.

Detailed questions and comments regarding the submission:

- What datasets are the language models pre-trained on? It is conceivable that the data sources themselves could be inherently biased in different ways (e.g. different news agencies having different biases). When evaluating the effect of specific model architecture on biases captured, it may be worthwhile to consider a more controlled setting where all models are trained on the same data source.

We have created a new table (Table 2) including information about the underlying training data and number of parameters for each of the analyzed language models. While the multilingual pre-trained language models have been trained on Wikipedia or Common Crawl, the monolingual English language models have been mostly additionally trained on BookCorpus and other datasets based on Common Crawl. BERTweet presents a notable exception as a monolingual language model for English that has been trained solely on tweets.

We agree that a setting where all models are pre-trained would provide more controlled results, and would enable isolated insights into the effect of model architecture. However, computational and environmental costs of these experiments would be vast. According to Bender et al. (2021), training a single BERT base model (without hyperparameter tuning) on GPUs was estimated to require as much energy as a trans-American flight. Additionally, the focus of our paper is on analyzing gender bias encoded in multilingual language models as they are deployed in real-life applications. Therefore, unfortunately we see this analysis as infeasible. However, in order to provide more controllable experimental setup, we have extended our analysis section with additional experiments. Further, we make a note in the main body of the paper that the term architecture encompasses here both the effect of model design and training data.

- Have you controlled for the time period in which the politicians are active? If so, which time periods do you consider? Not only that gender biases change over time, but also political sentiment in general. These changes can confound your result where some sentiment differences are not necessarily caused by gender differences.

We have queried names of politicians born from the 20th century onward, in order to prevent variability due to temporal changes. We agree that political sentiment changes faster than this time period, however language models in their training process have been potentially exposed to information from different periods. Additionally, information about the time period in which politicians were active might not always be accurate since many politicians are publicly active even if they currently don’t hold an official position. However, we wanted to include a temporal analysis, and have decided to split politicians into two groups, the ones born before and after Baby Boom. We hypothesize there might have been a shift in depictions of politicians who were born in that period and test this hypothesis in an additional experiment.

- The top 100 generated adjectives are considered for all languages but only the top 20 adjectives are considered for English. Is there a reason for this discrepancy?

In our experiments, we have established that the generated vocabulary for English is much more versatile than for the other languages (see Table 12). Already including top 20 adjectives and verbs for English results in a vocabulary set similar or bigger than for Spanish (the biggest vocabulary of out the remaining languages). Therefore, in order to decrease the computational costs and to maintain similar vocabulary sizes across languages, we decided to decrease the number of included words for English.

- In Table 4, what are the number of parameters associated with the model sizes (i.e large, xlarge, xxlarge)? Are these sizes consistent across the different architectures in terms of parameters counts?

We have created a new table (Table 2) including information about underlying training data and number of parameters for each of the analyzed language models to provide a better overview of analyzed language models. The number of parameters associated with the model sizes is similar for multilingual models, and for monolingual models with an exception of ALBERT.

Reviewer #2: The paper describes the problem of gender bias in language models and conducts a cross-lingual study to identify it. The gender-bias in language models is proxied through politicians' names and sentiment lexicon. The main claim is straightforward. The degree of gender bias is different in each language, and larger language models are not significantly more biased than smaller ones.

The problem studied in this paper has fundamental social implications and the paper has an interesting point of view. However, I have some concerns that require revision.

1) My main concern is about the choice of politicians' names. According to the paper, 250k names are retrieved from Wikidata. It is assumed that because these 250k names are for politicians and the fact that politicians have higher toxicity associations and bias in NLP models (Prabhakaran et al.), then the chosen names should represent the embedded gender bias in language models. However, I believe that not all of the names are relevant in all the 7 languages studied in the paper.

Although the BERT-based models are trained on Wikipedia, I doubt that a translated article about a politician on Wikipedia is enough to impose gender bias on the model. I strongly suggest that the experiments be run separately on a smaller set of names specific to each language to reduce the noise (e.g., for the Chinese language, only experiment on the Chinese politicians + a few internationally well-known politicians).

We follow reviewers' feedback and conduct a more in-depth analysis. We extend our experiments by three additional setups. First, we perform an analysis on a subset of 10k most popular politicians for each of the analyzed languages. Second, we conduct an analysis on a subset of names where for each language we examine words generated for politicians whose country of origin (i.e., their nationality) uses this language as an official language. Finally, we split our dataset and analyze words generated for politicians born before and after 1946. These experiments enable analysis in a more controllable setup.

2) Most of the experiments in the paper are done based on the frequency with which the top K words associated with a gender belong to a category (e.g., Negative sentiments, Positive sentiments, or a supersense). Although the results are interesting and should be reported, they are very similar to the ones in the previous work of Hoyle et al., 2019, except that now they are done cross-lingually and based on the politicians' data. I believe more experiments and tests should be done to assess gender bias and its differences across languages and language models. Exploring questions like which supersenses are more/less responsible for gender bias in different languages/LMs, and how systematically similar or different are the top gender-associated adjectives across languages can further strengthen the findings.

We agree that the created dataset enables a more in-depth analysis of gender bias in pre-trained language models. However, the available data on words with their respective supersenses covers solely 200 unique words. These words are not generated often enough by the monolingual English language models, and are not available for the remaining languages. Therefore, instead we have expanded upon analysis of differences in associations made for the most popular politicians, politicians that are native to the analyzed languaged, and analyzed generated words for politicians born before and after the end of World War II to incorporate a temporal analysis.

3) I faced some problems understanding section 5.1.2 and more specifically figure 3. How are you deriving the 100 largest-deviation adjectives for male and female genders? Isn't \eta(w, s) used for the deviation metric? If so how are you marginalizing the sentiment in this function? I think adding some detail about deriving the frequency of largest-deviation adjectives helps clarify this section.

We have expanded this section (now 6.1.2) with a more detailed description of the setup. We have conducted a two-sided permutation test in order to establish frequencies with which the 100 largest-deviation words are associated with positive and negative sentiment.

4) The paper proposes a critical point about the gendered adjectives and neutral words, and how they might mitigate the bias in language models trained on gendered languages (e.g., in Spanish). I am curious if there are ways to examine this proposal against the biases detected in the LMs corresponding to these languages. Some correlation analyses between the frequency of feminine/masculine inanimate words and the frequency of negative/positive adjectives associated with each gender might be one simple approach to this.

We agree that an analysis of gender bias encoded in multilingual language models based on the typology of a language, and presence of grammatical gender in particular, could provide valuable insights. However, out of the 7 analyzed languages only two do not have grammatical gender: English and Chinese. This fact strongly limits the scope and significance of this analysis in the current setup. Based on the results in our paper, we do not observe any association, however only a more extensive study over a larger number of languages could provide reliable results in this aspect.

My general argument is that the methodology used in the paper is statistically sound and evaluated in previous work, and the findings support the claim (although the claims themselves are not impressive), however, the experiments should be extended to deliver a more extensive set of findings.

We thank the reviewer for the valuable feedback. In the revised version of the paper, we have extended our experiments to provide a more in-depth analysis of gender bias towards politicians in multilingual language models and deliver more insights about the nature of bias encoded.

Reviewer #3: This paper explores gender bias toward politicians in transformer language models in a cross-lingual scenario. In particular, it investigates the adjectives/verbs associated with a large number of politicians in 7 languages, and measures deviations in sentiment toward male/female/'other' politicians in the respective language groups.

While the motivation and contribution of data set is laudable, as is the treatment of gender as non-binary. However, the paper (1) is not well-structured, at times repetitive and difficult to follow (esp for non-domain expert readers as attracted in PLOSONE); and (2) lacks motivation for several methodological decisions. Finally, the paper abstract/intro suggests a methodological contribution, while in section 4.2 it becomes clear that it simply re-uses a previously proposed method; and this method is not even described in detail.

We explicitly call our method simple and see it as a strength of our paper. The methods used in this paper to evaluate gender bias have been indeed proposed in prior work. We believe that the novelty of our paper lies in the newly proposed data generation strategy for evaluating (gender) bias in multilingual pre-trained language models, and the applicability of our method in multilingual scenarios.

In sum, I think this paper can make a worthwhile contribution by studying an important phenomenon at a broad scale, however, it needs careful rewriting and very careful motivation and validation of all steps of the approach. Please find detailed comments/suggestions below.

We thank the reviewer for the positive evaluation and valuable feedback.

Comments on paper quality / structure

- Section 3 dives right into templated probing methodology without motivating this approach. This compromises understandability of the paper, especially for an audience not focused on NLP (like PLOSONE). I think the paper would be more readable if Related Work was presented earlier on.

We have moved the Related Work section to precede the section on Gender Bias Detection, and the technical section on Dataset Generation. We have expanded upon our methodological choices in the experimental setup. We believe that these changes increase understandability of the paper.

- Similarly, for section 3.3. I think an illustration early on in the paper, of the overall approach (and goal) would help.

We have extended the explanation of our data generation process and have added an overview graph.

- Information is often presented in confusing order, e.g., a long list of models used (lines 133ff.) interrupting the explanation of generation methodology/POS filtering.

We agree that the previous structure of the paper was confusing. We have made a number of changes to increase the clarity of the paper. For instance, we have moved the list of analyzed language models, have added a detailed overview of the language models in an additional table (Table 2), and moved the related work section to an earlier point.

- The experiment setup is incomprehensible. A paper should be self-contained and "we conduct the same analysis as presented in [12]" (l. 244) is not sufficient.

We agree that a paper should be self-contained and provide enough information on the conducted analysis. Therefore, we have added a more detailed description of the experimental setup and its methodology.

Comments on methodology

- A discussion of the type of bias targeted would help, in particular (as far as I understand) it addresses bias based on properties of the *speaker* (i.e., language user) not bias toward properties of the entity being *spoken about* (i.e,. the politician). These dimensions have been disentangled and addressed separately (and jointly) in prior work (e.g., [1]).

We have expanded upon a discussion on gender bias in Section 3 (Gender Bias Detection). In our paper, we are interested in gender bias encoded in multilingual pre-trained language models *towards* politicians. Hence, following [1] we target *Gender of the Speaker*.

- The exact method of retrieving politicians names remains unclear. Was the process quality-checked? How well did it work for each language? How do you align politicians' names across languages?

We retrieve politician names for each politician using Wikidata. In Wikidata, we query all politicians and for each entry we retrieve the name in all 7 analyzed languages (upon availability). We present counts of politicians grouped by gender for each language in Table 1. Additionally, we have manually checked the correctness of gender annotations from Wikidata in order to ensure the quality of this information. Furthermore, we refer to our Ethical Considerations Section where we discuss quality assessment of collaborative knowledge bases such as Wikidata.

- The generation method still relies on syntactic cues, and will work better for languages in which the required bigrams are more plausible. E.g., for EN presumably all adjectives and most verbs will have very low probability. To what extent is the ranking meaningful? Was there any quality analysis across language?

We restricted the output of language models to be either adjective or verbs using annotations from UD treebanks which ensures that the generated words are descriptive of an entity of interest. We agree that there are differences in quality of the generated data across the analyzed languages. This can be seen in Table 12, the vocabulary generated for Hindi is only ⅕ of the number of unique lemmata generated for Spanish.

- Please motivate all parameters, including choice of languages, cut-off parameters (show the top20 / top100 words, rather than counts only)

We have selected the 7 analyzed languages based on their availability in several language models (m-BERT, XLM, and XLM-R), and typological diversity. In our experiments, we have established that the generated vocabulary for English is much more versatile than for the other languages (see Table 12). Already including top 20 adjectives and verbs for English results in a vocabulary set similar or bigger than for Spanish (the biggest vocabulary of out the remaining languages). Therefore, in order to decrease the computational costs and to maintain similar vocabulary sizes across languages, we decided to decrease the number of included words for English. We have added our motivation in the paper.

- An ablation study on comparable names (e.g., same number of subword tokens, comparable popularity) would have been interesting to disentangle LM perplexity from actual bias effects.

We have extended our experiments and have conducted an analysis of gender bias towards the most popular politicians for each of the analyzed languages.

- Similarly, the approach for creating sentiment lexica seems highly complicated. How did you choose the base models? Is there any evidence for the hypothesis that "combining a larger number of individual lexica with SentiVAE leads to more reliable results" (l. 173), i.e., how well did the most competitive base model perform in the tasks in Table 10?

Our aim was to ensure a high quality and coverage of vocabulary in the generated sentiment lexica. Moreover, we needed the lexica to include 3 sentiments (positive, neutral, and negative) and to have an equal scale for these sentiment values. Therefore, we chose the SentiVAE approach as an appropriate method for our approach.

- Table 10 does suggest significant drops for Chinese, Hindi and French; as well as a very low overall performance for Spanish. The conclusion that "the performance of over 72% is satisfactory and validates the lexicon approach" seems premature given that the bias detection method relies on quality sentiment classification, and the unconvincing results in Table 10.

We agree that there are performance drops for Chinese, and Hindi as well. We have toned down the description in the paper. However, these results are based on sentiment classification in more complex settings than solely evaluating one word as it is the case in our paper. In more complex tasks, such as sentiment classification of sentences and longer reviews, the sentiment lexicon approach might not be sufficient. In our paper, we treat these results as a lower bound performance in our single word scenario.

- Line 219 treats gender as binary, while the data set seems to be more nuanced (m, f, other). Please explain / unify.

As mentioned at the beginning of Section 5, due to the sparsity of the data for genders other than binary, it is infeasible to conduct the analysis with the latent-variable model. Therefore, we restrict ourselves in Section 5.2. to binary genders, while analyzing non-binary genders using PMI.

Comments on results / analyses

- Given that the contribution is a *cross-lingual* analysis, the corresponding results section is very shallow, the main take away being relative bias of small vs larger LMs.

- There's a missed opportunity of exploring in-depth research questions/analyses such as: are there systematic differences in bias across languages? How reliable is the method across languages (incl name selection, data generation, bias measurement)? How reliable is the method for high/low coverage politicians (potentially including politicians of the same vs. different native language).

In the revised version of the paper, following reviewers' feedback, we expand upon the initial results and offer additional experiments for a more in-depth analysis of gender bias towards politicians in multilingual language models.

[1] https://aclanthology.org/2020.emnlp-main.23.pdf

---

6. PLOS authors have the option to publish the peer review history of their article (what does this mean?). If published, this will include your full peer review and any attached files.

If you choose “no”, your identity will remain anonymous but your review may still be made public.

Do you want your identity to be public for this peer review? For information about this choice, including consent withdrawal, please see our Privacy Policy.

Reviewer #1: No

Reviewer #2: No

Reviewer #3: No

[NOTE: If reviewer comments were submitted as an attachment file, they will be attached to this email and accessible via the submission site. Please log into your account, locate the manuscript record, and check for the action link "View Attachments". If this link does not appear, there are no attachment files.]

While revising your submission, please upload your figure files to the Preflight Analysis and Conversion Engine (PACE) digital diagnostic tool, https://pacev2.apexcovantage.com/. PACE helps ensure that figures meet PLOS requirements. To use PACE, you must first register as a user. Registration is free. Then, login and navigate to the UPLOAD tab, where you will find detailed instructions on how to use the tool. If you encounter any issues or have any questions when using PACE, please email PLOS at figures@plos.org. Please note that Supporting Information files do not need this step.
