# Supplementary material for: Quantifying gender bias towards politicians in cross-lingual language models
Source: PLoS One. 2023 Nov 28;18(11):e0277640. doi: 10.1371/journal.pone.0277640 (PMC10684026; doi:10.1371/journal.pone.0277640)
Supplement: Attachment — Submitted filename: PLOS One Response.pdf [file pone.0277640.s009.pdf]

Dear PLOS ONE Academic Editor,

We would like to start by thanking the reviewers for their time and consideration in evaluating our work, and we are writing to ask you to examine a revised version of our manuscript. As a summary, our paper explores a simple method to probe pre-trained language models for gender bias, which we use to effect a multi-lingual study of gender bias towards politicians. We construct a dataset of 250k politicians from most countries in the world and quantify adjective and verb usage around those politicians' names as a function of their gender. We conduct our study in 7 languages across 6 different language modeling architectures.

We have considered reviewer 2's feedback and have extended the experimental section of the paper. We have conducted a more in-depth analysis of the words generated for the analyzed languages and language models in order to investigate semantic spaces in which words generated by language models are represented. In particular, we analyze word embeddings of the generated words for each of the three distinguished genders. To this end, we have employed PCA and a cluster analysis in order to identify semantic groups of generated words. We find that often the same words are being generated in association with different genders. However, based on previous results, words typically associated with the female gender are indeed generated more often to describe female politicians.

Further, we have expanded on Sections 4.3, 5.2, and 6.1-6.2 so that the paper is easier to follow for a broader type of audience than the NLP community. We have added more examples to clarify described steps of the analysis. We believe that these changes have clarified the methodology we have used.

We agree that the reliability of our experiments strongly relies on the quality of the sentiment lexica employed. Therefore, as mentioned above, we have extended Section 4.3 to include a more detailed explanation of the algorithm and our reasoning for using it in our analysis. The selected set of lexica that we used are all publicly available and have previously been verified on a sentiment classification task. Ultimately, we want to maximize the coverage of the words while retaining high quality of the lexicon used. However, due to disparities in their scales, we resort to the SentiVAE method to combine them. In the initial experiments, we have tested different combinations of lexica, evaluating them on the listed sentiment analysis datasets. Based on the results of this analysis, we exclude multilingual lexica for Hindi (described in the manuscript), as mentioned in the paper. The lexicon evaluation results for Spanish are indeed better than the results of a state-of-the-art supervised algorithm. Hence, although the results might seem relatively low, we can assume that they deliver results of as high a quality as it is currently possible. Further, the previously curated lexica were already created using human annotations and therefore, we do not see the added value in using more human annotations of the data. SentiVAE is solely a transformation of the lexica from their initial scale to be represented as a vector with values for the three sentiments, positive, negative and neutral.

In the manuscript, the sections marked in blue indicate revisions we have made for this submission, while red indicates the changes made for the previous one. Below, we respond to the specific questions the reviews have raised inline and we mark our comments with a distinguished font and a different color -- blue.

PONE-D-22-03986R1
Quantifying Gender Bias Towards Politicians in Cross-Lingual Language Models
PLOS ONE

Dear Dr. Stanczak,

Thank you for submitting your manuscript to PLOS ONE. After careful consideration, we feel that it has merit but does not fully meet PLOS ONE's publication criteria as it currently stands. Therefore, we invite you to submit a revised version of the manuscript that addresses the points raised during the review process.

Please submit your revised manuscript by Sep 22 2022 11:59PM. If you will need more time than this to complete your revisions, please reply to this message or contact the journal office at plosone@plos.org. When you're ready to submit your revision, log on to https://www.editorialmanager.com/pone/ and select the 'Submissions Needing Revision' folder to locate your manuscript file.

Please include the following items when submitting your revised manuscript:

- A rebuttal letter that responds to each point raised by the academic editor and reviewer(s). You should upload this letter as a separate file labeled 'Response to Reviewers'.
- A marked-up copy of your manuscript that highlights changes made to the original version. You should upload this as a separate file labeled 'Revised Manuscript with Track Changes'.
- An unmarked version of your revised paper without tracked changes. You should upload this as a separate file labeled 'Manuscript'.

If you would like to make changes to your financial disclosure, please include your updated statement in your cover letter. Guidelines for resubmitting your figure files are available below the reviewer comments at the end of this letter.

If applicable, we recommend that you deposit your laboratory protocols in protocols.io to enhance the reproducibility of your results. Protocols.io assigns your protocol its own identifier (DOI) so that it can be cited independently in the future. For instructions see: https://journals.plos.org/plosone/s/submission-guidelines#loc-laboratory-protocols. Additionally, PLOS ONE offers an option for publishing peer-reviewed Lab Protocol articles, which describe protocols hosted on protocols.io. Read more information on sharing protocols at https://plos.org/protocols?utm_medium=editorial-email&utm_source=authorletters&utm_campaign=protocols.

We look forward to receiving your revised manuscript.

Kind regards,

Yang Xu, PhD

Academic Editor

PLOS ONE

Additional Editor Comments:

Two reviewers provided comments on the revised manuscript. Although R1 found the paper acceptable, R2 raised a series of issues that should be addressed and discussed. Specifically, R2 suggested an in-depth analysis on the semantic representations of language models (that could have given rise to the results), a clearer

explanation of the methodology in Sec 5.2 (with the possible addition of an illustrative figure) and the working of SentiVAE in Sec 4.3, a more rigorous evaluation in Sec 4.3 e.g., using potentially human annotations and ablation, and a more detailed description of the supersets in Sec 6.1 and 6.1.2. These are reasonable comments, and I look forward to reading the next revision.

We thank the reviewers and the editor for their comments and valuable feedback throughout the reviewing process. We thank Reviewer 1 for the positive evaluation.

We have incorporated Reviewer 2's feedback by extending the experimental section of the paper. We have also conducted an analysis of the word embeddings of the generated words in order to identify semantic groups across languages and genders.

Further, we have expanded on Sections 4.3, 5.2, and 6.1-6.2, and believe that the resulting paper is clearer for a broader audience beyond NLP. We believe that these changes have clarified the methodology we used.

Since the selected lexica are already based on human annotations, and SentiVAE is solely a data transformation to bring different lexica to the same scale, we did not add human annotations to evaluate the sentiment lexica. We hope that, in light of our explanation, the reviewer would agree that this is not necessary.

We believe that the changes we have made to the manuscript have increased the readability of the paper and the extended experimental section has added merit to the analysis.

[Note: HTML markup is below. Please do not edit.]

Reviewers' comments:

Reviewer's Responses to Questions

Comments to the Author

1. If the authors have adequately addressed your comments raised in a previous round of review and you feel that this manuscript is now acceptable for publication, you may indicate that here to bypass the "Comments to the Author" section, enter your conflict of interest statement in the "Confidential to Editor" section, and submit your "Accept" recommendation.

Reviewer #1: All comments have been addressed

Reviewer #2: (No Response)

---

2. Is the manuscript technically sound, and do the data support the conclusions?

The manuscript must describe a technically sound piece of scientific research with data that supports the conclusions. Experiments must have been conducted rigorously, with appropriate controls, replication, and sample sizes. The conclusions must be drawn appropriately based on the data presented.

Reviewer #1: Yes

Reviewer #2: Yes

---

3. Has the statistical analysis been performed appropriately and rigorously?

Reviewer #1: Yes

Reviewer #2: Yes

---

4. Have the authors made all data underlying the findings in their manuscript fully available?

The PLOS Data policy requires authors to make all data underlying the findings described in their manuscript fully available without restriction, with rare exception (please refer to the Data Availability Statement in the manuscript PDF file). The data should be provided as part of the manuscript or its supporting information, or deposited to a public repository. For example, in addition to summary statistics, the data points behind means, medians and variance measures should be available. If there are restrictions on publicly sharing data—e.g. participant privacy or use of data from a third party—those must be specified.

Reviewer #1: Yes

Reviewer #2: Yes

---

5. Is the manuscript presented in an intelligible fashion and written in standard English?

PLOS ONE does not copyedit accepted manuscripts, so the language in submitted articles must be clear, correct, and unambiguous. Any typographical or grammatical errors should be corrected at revision, so please note any specific errors here.

Reviewer #1: Yes

Reviewer #2: No

---

6. Review Comments to the Author

Please use the space provided to explain your answers to the questions above. You may also include additional comments for the author, including concerns about dual publication, research ethics, or publication ethics. (Please upload your review as an attachment if it exceeds 20,000 characters)

Reviewer #1: The authors have adequately addressed comments raised in my previous review and have specifically improved clarity on their experimental setup as well as motivations behind various experimental decisions. An additional experiment has been performed to investigate the temporal effect of political sentiment.

To further improve the paper's clarity, it may be worthwhile to quickly elaborate on the decision to only include 20 adjectives in the main paper (e.g. selecting top 20 adjectives in English already offers a larger pool of adjectives) similar to what has been provided in the response letter.

We have now added an explanation to the paper of why we have only chosen 20 adjectives in English as opposed to 100 in other languages.

A sample of the dataset used has now been provided and can be accessed. The authors promise the public release of their data upon publication.

We thank Reviewer 1 for the helpful comments throughout the process of reviewing our paper and a positive evaluation of the paper in its current form.

Reviewer #2: The paper describes the problem of gender bias in language models and conducts a cross-lingual study to identify it. The gender bias in language models is proxied through politicians' names and sentiment lexicon. The main claim is straightforward. The degree of gender bias is different in each language, and larger language models are not significantly more biased than smaller ones.

Regarding my previous comments, the authors included new experiments for different sets of politician names. The results were like that of the initial experiment. However, I still believe that the paper lacks a more in-depth analysis. The current experiments show that language modes suffer from gender bias toward politicians, but this bias does not depend on the size of the model but on the language itself. Investigating how the representation of these languages varies in the semantic space generated by the language model could explain the current results.

Following reviewer 2’s feedback, we have added an additional experiment in which we conduct a cluster analysis of words generated for different gender categories in the analyzed languages and language models. These experiments enabled an in-depth analysis of semantic spaces generated by language models, as suggested by reviewer 2. We believe that this analysis has increased the value of the paper.

The methodology is almost completely borrowed from Hoyle et al., 2013. The paper, however, does not clearly explain how this method works in section 5.2. The current description is rather incomplete and difficult to follow without reading Hoyle et al., especially for those unfamiliar with NLP methodologies. I recommend rewriting this section to increase its readability. Adding an illustrative figure to summarize the approach would be helpful as well.

We have rewritten the methodology section and have elaborated on the model development steps. We believe that these additions clarify the method and make it readable independently of the Hoyle et al. 2019 paper.

The same thing applies to section 4.3. How does SentiVAE work? What are the differences in the chosen sentiment lexica? The generation of the multilingual sentiment lexica is a valid and important contribution of the paper and should be emphasized, but only a few paragraphs are dedicated to explain the approach and the results. Also, a more rigorous evaluation is needed for this section, especially for Hindi and Spanish, as the F1 scores are lower than the rest of the languages. Currently, the authors evaluate the generated sentiment lexicon based on the average sentiment of words in a sentence. Having a token-level approach toward sentiment analysis would indeed generate some noise. Using human annotations (even for a small section of the words) or conducting correlation analysis with the initial sentiment Lexia might be more accurate.

We thank the reviewer for recognizing the sentiment creation as one of the contributions of our work. We have elaborated now on the SentiVAE method and our choice of lexica in the main body of the paper to highlight the validity of our method.

As an ablation study, the authors could repeat the experiments in section 6 with one of the monolingual sentiment Lexia. This experiment clarifies whether using SentiVAE helps to identify gender bias in the language models or not.

We agree that the validity of our results depends on the reliability of the sentiment lexica. Therefore, as mentioned above, we have extended Section 4.3 to have a more detailed explanation of the algorithm and our reasoning for using it in our analysis. The selected lexica we used are all publicly available and have been previously verified in a downstream evaluation on a sentiment classification task. Ultimately, we want to maximize the coverage

of the words while retaining high quality of the lexicon used. However, due to disparities in their scales, we resort to the SentiVAE method to combine them. In the initial experiments, we have tested different combinations of lexica evaluating them on the listed sentiment analysis datasets. This analysis was a base to excluding certain lexica for Hindi, as mentioned in the paper. The lexicon evaluation results for Spanish are indeed better than the results of a state-of-the-art supervised algorithm. Hence, although they might seem relatively low, we assume that they deliver results of as high a quality as it is currently possible. Further, the previously curated lexica were created using human annotations and therefore, we do not see added value from using more human annotation on the data. SentiVAE is solely a transformation of the lexica from their initial scale to be represented as a vector with values for three sentiments, positive, negative and neutral. An ablation study that was suggested by the reviewer would not verify the lexicon since we do not have an explicit information of ground truth gender bias to compare it to report how much of it we detect using our lexicon.

I still recommend the authors to provide a more in-depth analysis that would go beyond the experiments and the results of the previous studies. The paper studies an important question and the authors have collected valuable data to investigate the question, but the experiments and the results provide only a surface-level analysis of this problem.

We're glad that the reviewer recognises the importance of our research topic. As indicated above, following their recommendation, we have extended the experimental section of the paper. We believe that the new results increase the value of our paper and provide valuable insights about the nature of bias encoded in language models across the analyzed languages.

The readability of some parts, especially the methodology and the experiments, should be improved as well.

We have rewritten the methodology section and added more detailed explanations of models used. We have added further experiments which we describe above.

Writing comments:

In section 6.1., the authors could provide a description of the supersets used and the set of words each contains.

We have added a description of the supsersets together with words they include in the Appendix.

I find it difficult to understand the first paragraph of section 6.1.2. Could you provide some examples for each step mentioned in this section?

We have rewritten this part of the paper and added a step by step explanation, and examples.

---

7. PLOS authors have the option to publish the peer review history of their article (what does this mean?). If published, this will include your full peer review and any attached files.

If you choose "no", your identity will remain anonymous but your review may still be made public.

Do you want your identity to be public for this peer review? For information about this choice, including consent withdrawal, please see our Privacy Policy.

Reviewer #1: No

Reviewer #2: No
